# The T allele of lysyl oxidase-like 1 rs41435250 is a novel risk factor for pseudoexfoliation syndrome and pseudoexfoliation glaucoma independently and through intragenic epistatic interaction

**Published:** 2013-09-16

**Authors:** Dalia Guadarrama-Vallejo, Antonio Miranda-Duarte, Juan Carlos Zenteno

**Affiliations:** 1 Department of Genetics–Research Unit, Institute of Ophthalmology “Conde de Valenciana” Mexico City, Mexico; 2 Department of Genetics, National Rehabilitation Institute, Mexico City, Mexico; 3 Department of Biochemistry, Faculty of Medicine, National Autonomous University of Mexico (UNAM), Mexico City, Mexico

## Abstract

**Purpose:**

Two coding single nucleotide polymorphisms (SNPs) in lysyl oxidase-like 1 (*LOXL1*) are major genetic risk factors for pseudoexfoliation syndrome (XFS) and pseudoexfoliation glaucoma (XFG) in diverse populations. However, recent conflicting results suggest that the currently known disease-associated missense variants R141L and G153D are not causal and that they may be proxies for other unknown functional *LOXL1* variants. The purpose of this study was to investigate the possible association of XFS/XFG with a novel *LOXL1* exonic variant.

**Methods:**

Genotypes of the synonymous coding *LOXL1* SNP rs41435250 (p.A310A) were identified with direct sequencing. A case-control study was conducted with 115 unrelated Mexican patients with XFS/XFG (43 XFS/72 XFG) as well as 130 control subjects. Allele frequencies, genotype frequencies, and Hardy–Weinberg equilibrium were assessed with the HaploView software. A probable intragenic epistasis effect was assessed by comparing the frequencies of the rs41435250 alleles among a subset of 51 patients with XFS/XFG without the high-risk TT genotype at *LOXL1* intronic rs2165241 and the control group.

**Results:**

The T allele of the exonic SNP rs41435250 was more frequent in patients with XFS/XFG than in controls (odds ratios [95% confidence intervals]=2.0 [1.1–3.6]; p=0.01). Interestingly, the strength of association with the rs41435250 T allele was strongly increased (odds ratio [95% confidence intervals]=4.9 [2.7–9.1]; p=0.00000005) in the subgroup of subjects without the risk genotype at rs2165241.

**Conclusions:**

Our results indicate that allele T of rs41435250 is a novel risk genetic factor for XFS/XFG development in our population and points toward the possibility of a *LOXL1* intragenic epistatic effect between rs41435250 and rs2165241. Functional studies are needed to investigate if the synonymous p.A310A mutation could affect messenger ribonucleic acid stability and thus LOXL1 enzymatic activity.

## Introduction

Glaucoma, the leading cause of irreversible blindness in the world, is defined as a group of progressive optic neuropathies in which the axons in the optic nerve are injured, retinal ganglion cell numbers are reduced, and vision is gradually and permanently lost. The most common identifiable cause of secondary open-angle glaucoma (OAG) is pseudoexfoliation syndrome (XFS), a systemic age-related disease characterized by an abnormal accumulation of fibrillar material in ocular and extraocular tissues [1], and which affects approximately 10%–20% of Caucasian individuals over the age of 60 [2].

XFS is responsible for as high as 20%–60% of OAG cases in many regions of the world, including Europe, Asia, and Africa [3,4]. It has been estimated that the risk of XFS syndrome developing to exfoliation glaucoma (XFG) over a period of 15 years is about 60% [5]. At the clinical level, XFG has a worse prognosis than primary OAG, shows a poor response to medical treatment, and has a rapid progression of optic neuropathy [6].

Extensive evidence indicates that genetic factors play a key role in the pathogenesis of XFS/XFG, including ethnic differences in the prevalence of the disease, positive family history, and increased risk in relatives and twin studies [4]. In 2007, a genome-wide association study in Icelandic and Swedish individuals with XFG identified a strong association between the disease and single nucleotide polymorphisms (SNPs) rs1048661 (R141L), rs3825942 (G153D), and rs2165241 (intronic) of the Lysyl oxidase-like (*LOXL1*) gene, at 15q22 [7]. The protein encoded by *LOXL1* is a member of the lysil oxidase family involved in the cross-linking of collagen and elastin in the extracellular space [8,9]. In recent years, several replication studies in populations from North America [10-12], Europe [13,14], Asia [15-18], and Australia [19] have subsequently confirmed the solid association of *LOXL1* polymorphisms with XFS/XFG, indicating that *LOXL1* is the major genetic risk factor for disease development. Intriguingly, in some populations different alleles of each of the *LOXL1* XFS/XFG high-risk SNPs have been associated with the disease [20], suggesting that still-unidentified genetic or environmental factors may contribute to disease pathophysiology.

In a recent study, we demonstrated that the T allele of the *LOXL1* intronic rs2165241 SNP conferred higher risk for XFS/XFG in a Mexican population, rather than the worldwide “high-risk” G allele of rs3825942 [21]. To gain further insight into the involvement of *LOXL1* in XFS/XFG pathogenesis in our population, in the present study we searched for the association of additional variants in this gene in a sample of Mexican subjects with the disease. Our results indicate that the exonic rs41435250 SNP is strongly associated with XFS/XFG and that the strength of this association is modified by the patient’s genotype at the intronic variant rs2165241. Our results indicate that rs41435250 is a novel risk genetic factor for XFS/XFG and suggest a model of intragenic epistasis of *LOXL1* SNPs in the predisposition to develop the disease.

## Methods

### Study subjects

A total of 115 patients with XFS/XFG and 130 control individuals of Mexican Mestizo origin were evaluated at the Conde de Valenciana Institute of Ophthalmology in Mexico City. A Mexican Mestizo is defined as a person who was born in Mexico, has a Spanish-derived last name, and has a family of Mexican ancestors going back to the third generation [22]. No familial history of XFS/XFG was reported by any subject in either group. All subjects underwent detailed ophthalmological examinations, including slit-lamp biomicroscopic assessment, applanation tonometry, gonioscopy, dilated inspection of the lens, and funduscopy. Subjects with XFS were defined as those with clinical evidence of exfoliation material at the pupil margin, anterior lens surface, or other anterior segment structures, with intraocular pressure of less than 21 mmHg and no clinical evidence of glaucomatous optic neuropathy [12]. Subjects with XFG were defined as those with clinical evidence of XFS and glaucomatous optic neuropathy (defined as loss of the neuroretinal rim with a vertical cup:disc ratio of greater than 0.7) with compatible visual field loss [12]. Controls were recruited from the General Ophthalmology and Retina departments of our hospital according to the following criteria: (1) subjects aged 55 years or older evaluated for refractive errors, (2) no signs of XFS or XFG, (3) no glaucomatous changes in the optic disc, and (4) normal visual fields and intraocular pressure. Written informed consent was obtained from all participants, the study protocol was approved by the hospital ethics committee, and the study was performed according to the tenets of the Declaration of Helsinki.

### Lysyl oxidase-like 1 polymerase chain reaction amplification and sequence analysis

The entire coding sequence (seven exons) of *LOXL1* was screened using PCR amplification and direct nucleotide sequencing. Primers are shown in Table 1. Each 25 μl PCR amplification reaction contained 1X buffer, 200 ng of genomic DNA, 0.2 mM of each deoxynucleotide triphosphate, 2U *Taq* polymerase, 1 mM of forward and reverse primers, and 1.5 mM of MgCl_2_. PCR products were analyzed in 1.5% agarose gels, from which the bands with the amplified templates were excised and the DNA subsequently purified with the help of the Qiaex II kit (Qiagen, Hilden, Germany). Direct automated sequencing was performed with the BigDye Terminator Cycle Sequencing kit (Applied Biosystems, Foster City, CA), adding about 15 ng of template DNA in each reaction and using a temperature program that included 25 cycles of denaturation at 97 °C for 30 s, annealing at 50 °C for 15 s, and extension at 60 °C for 4 min. Samples were analyzed in an ABI Prism 3130 Genetic Analyzer (Applied Biosystems).

**Table 1:** Primer sequences for PCR amplification of *LOXL1* gene.

Exon	Primer sequence (5′-3′)	Annealing temperature (C°)	Amplicon size (bp)
1AF	TTCCTCAGAGGCAGGTCTGT	56.7	681
1AR	CGTACTCTGAGCCCGAGTTG		
1BF	AGGGGTCACCATGGCTCT	56.7	700
1BR	GGGCTGGTAGGGGTAGATGA		
1CF	CCTTCGTCAGCCAGTACGAG	56.7	652
1CR	GCCTCCAGGAAGTTCTAAGGA		
2F	GGAGGTCTCTGGGCATTAGC	58.7	294
2R	TCACTGATGAAACGGTCAGG		
3F	TCTTCAGGGACAAGGAGTGG	56.6	357
3R	AGTGCTCATGGTGGGAGG		
4F	CAGGGAAGACTAGGCCCTCT	58.7	324
4R	CTGTGAGCAGAGCTGAGTGG		
5F	GAAACAAGCAGCATCACAGC	54.6	220
5R	GGCTGAAGCTTCTTTCAGGA		
6F	TCTGGTGAGCAGTTGAGGTG	56.6	205
6R	GGTGGTGGGCAAACTCTTAC		
7F	CCCTCATTGACCCACTGTCT	56.6	399
7R	CCAGGCCCAAACTAGCTG		

Annealing temperatures and amplicon sizes are also indicated.

### Statistical analysis

Allelic and genotypic frequencies as well as Hardy–Weinberg equilibrium were assessed with HaploView 4.0 software (Daly Lab, Broad Institute, Cambridge, MA). Statistical analysis included comparisons of continuous variables with the Student *t* test while the chi-square test was applied for categorical variables. To determine the risk magnitude, odds ratios (ORs) and their 95% confidence intervals (95% CI) were reported. The alpha level was 0.05, and the STATA ver.10.0 statistical software package (StataCorp LP, College Station, TX) was used for calculations.

## Results

A total of 115 patients with XFS/XFG and 130 control individuals were genotyped. Patient groups included 72 cases with both XFS and XFG and 43 cases with XFS without glaucoma (Table 2). The mean age at recruitment was 75.1±7.8 years in the cases and 71.9±8.1 years in the controls (p=0.002). The gender distribution between the cases and the controls was not significantly different (p=0.43). The age range in the patients group was 50–95 years (62–95 years for XFS; 50–90 years for XFG), while the age range in the control group was 55–88 years.

**Table 2:** Demographic characteristics of XFS/XFG patients and controls.

Parameters	Patients (n=115)	Controls (n=130)	p
Females (n, %)	78 (67.8)	82 (63.1)	0.43
Age (Mean SD)	75.1±7.8	71.9±8.1	0.002
XFG	72 (62.6)		
XFS	43 (37.4)		

### Lysyl oxidase-like 1 sequence analysis

**Figure 1:**
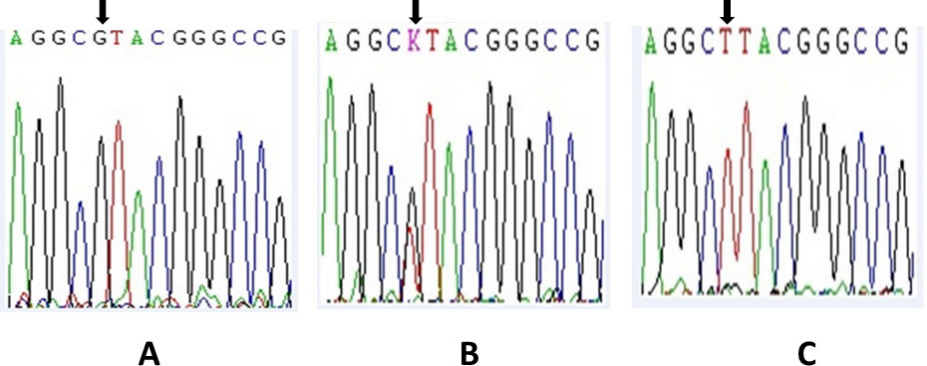
Partial nucleotide sequence of lysyl oxidase-like 1 gene exon 1 showing single nucleotide polymorphism rs41435250 in deoxyribonucleic acid from individuals with pseudoexfoliation syndrome or pseudoexfoliation glaucoma. The arrow indicates the polymorphic nucleotide and sequence traces for homozygous G, heterozygous G/T , and homozygous T samples are shown in **A**, **B**, and **C**, respectively.

To identify polymorphic variants in *LOXL1*, the entire coding sequence of this gene was initially sequenced in a subgroup of 15 subjects with XFS. After sequencing, the only additional variant identified (besides the previously known rs1048661 (R141L), rs3825942 (G153D), and intronic rs2165241) was SNP rs41435250 (c.960G>T, pAla320Ala), located at exon 1 of *LOXL1* (Figure 1). Afterward, all remaining patients and the 130 control subjects were genotyped for rs41435250 with direct sequencing.

### rs41435250 allelic and genotypic frequencies

The genotypic distributions of rs41435250 in the control group followed Hardy–Weinberg equilibrium (p=0.17). The allelic and genotypic distributions of this SNP and the evaluation of its association with XFS/XFG are shown in Table 3. Allelic association analysis showed that there were significant differences in the allelic distributions of rs41435250 between the two groups. As shown, the T allele of this SNP was significantly more frequent in the patients with XFS/XFG than in the controls (OR [95% CI]=2.0 [1.1–3.6]; p=0.01). The TT genotype was not significantly associated with the disease condition (OR [95% CI]=2.2 [0.4–12.8]; p=0.3).

**Table 3:** Allelic and genotype association testing results of rs41435250 for Mexican XFS/XFG patients and controls.

rs41435250	Patients (n=115)	Controls (n=130)	OR (95% CI)	P
Allele				
T	35 (15.2)	21 (8.1)	2.0 (1.1 – 3.6)	0.01
G	195 (84.8)	239 (91.9)	0.49 (0.3 – 0.9)	0.01
Genotype				
TT	4 (3.5)	2 (1.5)	2.2 (0.4 – 12.8)*	0.3
GT	27 (23.5)	17 (13.1)	1.9 (1.0 – 3.9)*	0.05
GG	84 (73.0)	111 (85.4)	0.5 (0.2 – 0.9)*	0.02

Abbreviations: n=number of subjects; OR=odds ratio; CI=confidence interval. *Adjusted by gender and age.

From the group of 115 patients with XFS/XFG, we analyzed a subset of 51 individuals who were proven to be negative for the TT high-risk genotype (i.e., CC or CT) at the intronic rs2165241 SNP in our previous study [21]. When their allelic and genotypic frequencies were compared with the entire group of 130 control subjects, the strength of association with the T allele of rs41435250 was strongly increased (OR [95% CI]=4.9 [2.7–9.1]; p=0.00000005; Table 4). In agreement, the G allele and the GG genotype at rs41435250 exhibited a clear significant protective effect against XFS/XFG (Table 4).

**Table 4:** rs41435250 allelic and genotypic association testing results in a subgroup of 51 XFS/XFG patients who were negative for the TT risk genotype at rs2165241 and 130 controls.

rs41435250	Patients (n=51)	Controls (n=130)	OR (95% CI)	P
Allele				
T	31 (30.0)	21 (8.1)	4.9 (2.7 – 9.1)	5E-08
G	71 (70.0)	239 (91.9)	0.2 (0.1 – 0.4)	5E-08
Genotype				
TT	4 (7.8)	2 (1.5)	5.5 (0.9 – 32.1)*	0.06
GT	23 (45.1)	17 (13.1)	5.1 (2.4 – 11.0)*	0.0001
GG	24 (47.1)	111 (85.4)	0.2 (0.08 – 0.3)*	0.00001

A marked increased risk associated to the rs41435250 T allele was observed. Abbreviations: n=number of subjects; OR=odds ratio; CI=confidence interval. *Adjusted by gender and age.

Finally, when the allelic and genotyping frequencies were separately analyzed between the two sub-phenotypes, i.e., subjects with XFS without glaucoma and subjects with XFS/XFG, the rs41435250 T allele risk magnitude was maintained in the patients with XFS while a marginally statistically significant risk was observed in the XFS/XFG group (OR [95% CI]=1.8 [0.9–3.5]; p=0.06; Table 5).

**Table 5:** rs41435250 allelic and genotypic association testing results for XFS patients and XFS-XFG patients.

XFS/XFG	Patients (n=72)	OR (95% CI)*	P
Allele (rs41435250)			
T	20 (13.9)	1.8 (0.9 – 3.5)	0.06
G	124 (86.1)	0.5 (0.3 – 1.0)	0.06
Genotype			
TT	2 (2.8)	1.8 (0.1 – 25.1)	0.5
GT	16 (22.2)	1.9 (0.8 – 4.3)	0.09
GG	54 (75.0)	0.5 (0.2 – 1.1)	0.06

XFS	Patients (n=43)	OR (95% CI)*	P
Allele (rs41435250)			
T	15 (17.4)	2.4 (1.2 – 4.9)	0.01
G	71 (82.6)	0.4 (0.2 −0.8)	0.01
Genotype			
TT	2 (4.6)	3.1 (0.2 – 43.9)	0.2
GT	11 (25.6)	2.3 (0.8 – 5.8)	0.05
GG	30 (69.8)	0.4 (0.2 – 0.9)	0.02

*Compared with 130 controls

## Discussion

The prevalence of XFS/XFG varies widely across populations, ranging from 20% to 25% in the Scandinavian countries of Iceland and Finland [23] to less than 1% in Asian populations [24-26]. These ethnic-related differences in prevalence figures suggest that several genetic and/or environmental factors may play a role in XFS development. SNP variants at *LOXL1* are the most important genetic risk factor for developing XFS/XFG in distinct ethnic groups [4]. However, independent replication of these findings in additional populations is important for further delineation of the molecular basis of XFG and for the possible identification of secondary genetic factors that can also lead to the disease. A current limitation in XFS/XFG-*LOXL1* association studies is that, typically, most replication analyses have been performed in Asian, Caucasian, and European populations, and some ethnic groups are underrepresented [20].

Despite the consistent association of *LOXL1* variants with XFS/XFG in numerous ethnic groups worldwide, several conflicting individual allelic associations have been identified. For example, in all of the Japanese studies conducted to date, the disease-associated allele of rs1048661 was T, instead of G as seen in Caucasian or Scandinavian studies [15,27,28]. In addition, in black South Africans the risk allele for rs3825942 was A instead of G [29], which is the disease-associated allele in all other analyzed XFS/XFG populations. Our previous study in a Mexican population showed that instead of the “universal” risk (G) allele of rs3825942, the T allele of the intronic SNP rs2165241 conferred higher risk for XFS/XFG development in our population [21]. This finding motivated us to search for additional *LOXL1* variants that could account for disease risk. Thus, we identified that the T allele and the TT genotype of the exonic SNP rs41435250 were significantly associated with the disease with an OR of 2. Then, we speculated that our previously identified patients with XFS/XFG without the high-risk TT genotype at intronic rs2165241 might have an elevated frequency of the T allele at rs41435250. After 51 patients with XFS/XFG negative for the TT genotype at rs2165241 were compared with a group of 130 controls, the strength of association increased strikingly (OR=4.9; p=0.00000005). In agreement, the G allele and the GG genotype of rs41435250 were clearly associated with a protective effect against the disease.

Our data indicate that, at least in our population, the T allele of *LOXL1* rs41435250 is a high-risk factor for XFS/XFG. To the best of our knowledge, this variant has been analyzed only in Saudi Arabian [29] and South African [30] populations, in whom it was monoallelic (G allele) in individuals with XFG/XFS and control individuals. Our results add to previous observations concerning the occurrence of significant interethnic variations in allelic frequencies (and consequently in disease association strength) of XFS/XFG-risk *LOXL1* variants. A weakness of our study is that a formal linkage disequilibrium analysis between rs41435250 and rs2165241 was not performed, and thus, additional replication of our findings is needed to demonstrate an intragenic epistatic effect.

The T allele of rs41435250 codes for a synonymous p.Ala310Ala (GCG to GCT) mutation at the conserved N-terminal domain of the LOXL1 proenzyme. Since this domain is important for the correct enzyme activation and substrate recognition and binding, a polymorphism in this region may affect enzyme function [9]. Although in general it is agreed that synonymous SNPs are not related to disease based on the assumption that these are “silent” and do not affect protein expression or function, there is also evidence that they can alter processes as messenger ribonucleic acid (mRNA) stability, kinetics of translation, and splicing. In conjunction or individually, these mechanisms can lead to changes in protein amount, structure, and/or function [31]. For example, there is clear evidence that synonymous codons are not used randomly (codon usage bias) and that preferred codons correlate strongly with the relative abundance of the corresponding transfer ribonucleic acid [31]. Codon usage is one way in which kinetic control of translation elongation is imposed. The speed of translation is often important for accurate folding of the protein and to reduce misfolded or aggregated molecules, which have adverse consequences. Synonymous mutations that affect codon usage can disrupt this process [32]. The kinetics of protein folding could be influenced by codon bias because the rate of translation would be rapid over mRNA stretches that use frequent codons and slower when rare codons are used [33]. In this context, it is remarkable that only two out of 54 alanines found in the LOXL1 protein use codon GCT, which is the synonymous alanine codon resulting from the T risk allele in rs41435250.

Although the association of XFS/XFG with the T allele of *LOXL1* rs41435250 was clearly evident in our patients, this association was markedly stronger in the patients without the TT high-risk genotype at intronic rs2165241 in this same gene [21], suggesting an intragenic *LOXL1* epistatic effect. Intragenic epistasis occurs when a variation in one location of a gene influences the phenotype differently depending on other variations within the same gene [34]. To our knowledge, this is the first study suggesting such an effect for XFS/XFG-*LOXL1* high-risk variants. However, additional studies are required to support this possibility.

Finally, case-control genetic association studies in an admixed population are susceptible to genetic confounding due to population stratification. Thus, ancestry informative markers should be analyzed to avoid bias [35]. In our study, however, the source population from which the controls were sampled did not differ from that of the cases and the subjects in both groups originated from the same geographic region of the country. Thus, spurious associations resulting from the presence of genetically different strata in our study sample are unlikely.

In conclusion, we identified SNP rs41435250 as a novel risk genetic factor for XFS/XFG development and presented evidence of a possible *LOXL1* intragenic epistatic effect between rs41435250 and rs2165241. Functional studies are needed to investigate if the synonymous p.A310A mutation could affect mRNA stability and thus LOXL1 enzymatic activity.
